# Are persons with fibromyalgia or other musculoskeletal pain more likely to report hearing loss? A HUNT study

**DOI:** 10.1186/s12891-016-1331-1

**Published:** 2016-11-16

**Authors:** Magne Stranden, Håvard Solvin, Egil A. Fors, Linn Getz, Anne-S. Helvik

**Affiliations:** 1 Department of Public Health and General Practice, Faculty of Medicine, Norwegian University of Science and Technology (NTNU), Trondheim, Norway; 2 St. Olav’s University Hospital, Trondheim, Norway; 3 Norwegian National Advisory Unit on Aging and Health, Vestfold Health Trust, Tønsberg, Norway; 4 Harald Haarfagres gate 2, Trondheim, NO-7041 Norway

**Keywords:** Fibromyalgia, Subjective hearing loss, Musculoskeletal pain, Central sensitivity syndrome, Chronic activation theory of stress, Allostatic load

## Abstract

**Background:**

Leading theories about the pathogenesis of fibromyalgia focus on central nervous dysregulation or sensitization, which can cause altered perception. There is growing evidence that fibromyalgia involves altered perception not only of pain, but also other sensory stimuli. On this basis, we investigated whether individuals with fibromyalgia are more likely to report subjective loss of hearing, adjusted for audiometrically measured loss of hearing, compared to persons without any musculoskeletal pain disorders. In addition, we studied persons with other musculoskeletal pain than fibromyalgia and persons who did not have any musculoskeletal pain.

**Methods:**

The study includes 44 494 persons from the second health survey in Nord-Trøndelag (HUNT2) who had undergone audiometry and answered a comprehensive questionnaire that mapped fibromyalgia, musculoskeletal pain at various sites and subjective hearing loss. Respondents with other musculoskeletal pain problems than fibromyalgia were divided into two groups with respectively localized and widespread musculoskeletal pain. Data were analyzed with logistic regression models adjusting for age, education, anxiety, depression and hearing thresholds.

**Results:**

In adjusted analysis, individuals with fibromyalgia had increased likelihood to report subjective hearing loss, compared to persons without fibromyalgia or other musculoskeletal pain (OR 4.578, 95% CI 3.622–5.787 and OR 4.523, 95% CI 3.077–6.647 in women and men). Furthermore, people with local and widespread musculoskeletal pain not diagnosed with fibromyalgia, also had increased likelihood to report subjective hearing loss, compared to people with no musculoskeletal pain. This relationship was greater for widespread pain than for localized pain (OR 1.915, 95% CI 1.627–2.255, and 1.796, 95% CI 1.590–2.029, in women and men with local musculoskeletal pain and OR 3.073, 95% CI 2.668-3.539, OR 3.618, 95% CI 3.225–4.058, in women and men with widespread pain, respectively).

**Conclusions:**

Our findings are consistent with the hypothesis that fibromyalgia is related to a general dysregulation of the central nervous system. The same might also be the case for other local and, in particular, other widespread, musculoskeletal pain.

## Background

Prolonged pain from the musculoskeletal system and other symptoms currently associated with the diagnosis fibromyalgia have been described since ancient times [1, 2]. In the 1500s such symptoms was termed “rheumatism” [3] and in the 1700s “muscular rheumatism” [3]. From the early 1900s the term “psychogenic rheumatism” was presented, although it was assumed to be caused by muscular inflammation and preferentially named “fibrositis” [4–6]. Eventually, in 1976, the term fibromyalgia was coined [7], as the symptoms were no longer considered to have an inflammatory cause, i.e. the past prevailing paradigm since Gowers in 1904 [3, 8]. The etiology and pathogenesis is since then often characterized as medically unexplained [9]. However, fibromyalgia may be considered as a “discrete diagnosis or as a constellation of symptoms characterized by central nervous system pain amplification with concomitant fatigue, memory problems, and sleep and mood disturbances”[10]. The estimated prevalence of fibromyalgia in the general population varies globally between approximately 2 and 11%, depending on the population and study design [10, 11]. The prevalence is higher in women than men (9:1), and increasing with age [12]. The diagnosis has until recently been determined by clinical examination according to the ACR 1990 criteria, in which the patient must have pain in all of the body’s four quadrants plus axial pain, and at least 11/18 predefined tender points, triggered by a pressure of a maximum of 4 kg/cm^2^ [13]. In addition to being a chronic, widespread musculoskeletal pain condition without a well-defined cause, fibromyalgia is often accompanied by non-specific symptoms and comorbidities [14–16]. These include symptoms such as fatigue, memory and concentration problems [17], sleep disturbances, stomach ache, depressive symptoms and headache [10, 18, 19], and disorders like irritable bowel syndrome (IBS), chronic fatigue syndrome (CFS/ME), interstitial cystitis (IC) and temporomandibular disorder (TMD) [8].

Due to the high prevalence of symptoms and comorbidities associated with fibromyalgia, researchers in various milieus have started to view fibromyalgia and related conditions as potentially explained by the same mechanisms [20]. The prevailing view is that they represent a similar, altered central neural processing of perceptive stimuli, rather than organ-specific pathology. One suggested term to cover such a neural dysregulation condition is “centralized sensitization syndrome” (CSS) [21, 22]. Other research groups have launched concepts and theories, which are theoretically in good coherence with the notion of central sensitization. These include sustained arousal [23], the Cognitive Activation Theory of Stress (CATS) [24] and allostatic overload [25]. Currently, these concepts exist more or less in parallel, and no consensus exists about the mechanisms [26, 27]. However, the diagnostic criteria for fibromyalgia have since 2010 been adapted to accommodate the frequent occurrence of associated symptoms and comorbidities [18]. The diagnosis can now be established by therapist interviews and self-reports by summing the pain localizations (0–19) in a “widespread pain index” (WPI) plus adding a 0–12 ranged “symptom severity” score (SSS) considering the comorbid symptoms mentioned above. This completes the ACR 2010 fibromyalgia criteria and the later 2011 “Fibromyalgia survey criteria” by a sum score of maximum 31 points where the fibromyalgia diagnosis is defined by a 12 point cut-off score [18, 19].

Recently, researchers have taken interest in how patients with fibromyalgia experience hearing. One study has found increased incidence of reported subjective hearing loss among persons with fibromyalgia, compared to individuals with inflammatory rheumatic disorders [28]. Fibromyalgia has also been associated with hypersensitivity to noise [29]. These findings are interesting in light of the theories concerning central sensitization, and are compatible with studies of cognitive dysfunction and memory problems in fibromyalgia and chronic widespread pain [17]. Another study has found poor correlations between subjective and objective hearing loss in patients with three or more medical unexplained symptoms, but that study did not include fibromyalgia per se [30]. It is thereby still unclear whether persons with fibromyalgia are more likely to report hearing loss than others. According to the theory of sustained arousal, one might hypothesize that if persons with fibromyalgia can be shown to experience auditory disturbances in addition to the previously documented problems with cognitive function and memory [17], similar auditory disturbances might also be found in persons with widespread muscular pain.

Based on The Nord-Trøndelag Health Study part 2 (HUNT2) and Nord-Trøndelag Hearing Loss study (NTHLS), the aim of the present study was to explore if people who report fibromyalgia or other musculoskeletal pain are more likely to have subjective hearing loss, compared to controls without such problems. More specifically, our research question is:

Are persons with fibromyalgia or other musculoskeletal pain, widespread or localized, more likely to report subjective hearing loss than persons without fibromyalgia or other pain, when adjusting for measured hearing thresholds, age, gender and education, as well as depression and anxiety?

## Method

### Study design and participants

Data from HUNT 2 (1995–7) and the NTHLS were used in a retrospective cross-sectional study. HUNT2 and NTHLS are questionnaire-based, but hearing loss was assessed by audiologists who used mobile research units to measure audiometry.

**Fig. 1:**
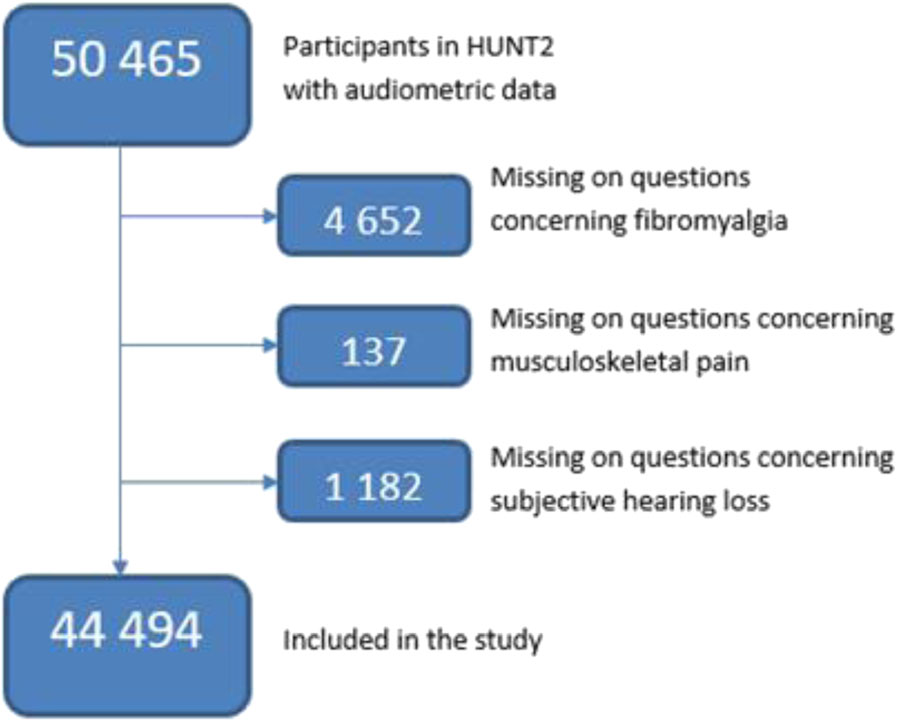
Inclusion form

In total, 66 140 adults, age ranging from 20 to 101 years participated in HUNT2. Median age was 48, and mean age 50.2 years [31]. Hearing tests were available for 50 465 participants. Persons with missing data on fibromyalgia, musculoskeletal pain and subjective hearing loss were excluded from the present study (Fig. 1). All participants in HUNT 2 and NTHLS provided informed, written consent to participate in research studies.

### Measures

#### Fibromyalgia

Fibromyalgia was assessed with the question: “Has a physician ever said that you have had fibromyalgia (fibrositis/chronic pain syndrome)?” (HUNT 2 Q1) [32], with response alternatives “yes” or “no”. An affirmative answer to this question classified respondents as having fibromyalgia. It is not known to what extent physicians used the tender-point criteria when establishing the diagnosis [13].

#### Other musculoskeletal pain

Other musculoskeletal symptoms than fibromyalgia were evaluated by self-report questions from the HUNT2 Q1 questionnaire [32]. Similar questions have shown good sensitivity and reproducibility in earlier studies [33–35]. The initial question was: “Have you during the past year suffered from pain and/or stiffness in muscles and limbs that have lasted for at least three consecutive months? If so, where did you have these ailments?” Subsequently the participant could select between 10 different localizations [36]. Affirmative answers to one or more of the questions concerning musculoskeletal pain or stiffness, and not “yes” to the fibromyalgia question, were categorized as “having other musculoskeletal pain than fibromyalgia”.

#### Widespread or localized pain

Persons with muscular and/or skeletal problems in three or more localizations were categorized as having “widespread musculoskeletal pain” [37]. Those who had one to two localizations for musculoskeletal pain were categorized as having “localized musculoskeletal pain”.

#### Subjective hearing loss

Subjective hearing loss (dependent variable) was assessed by the following questions and follow-up questions [32]: “Do you have any long-term illness, injury or suffering of physical or psychological nature that impairs your functioning in your daily life?”. Then, if yes, “Impaired hearing?” and “How much would you say that your functions are impaired?” with grading options 0–3 (0 = not impaired, 1 = slightly impaired, 2 = mediocrely impaired and 3 = seriously impaired). Options 1–3 were interpreted as subjective loss of hearing.

#### Audiometry

Audiometry measured the hearing threshold for air conduction. It was performed by trained personnel under good conditions [31, 38], and the method test-retest reliability is high [39]. The audiometry was performed automatically with earmuffs connected to a PC. In cases where the participant was not able to conduct the test automatically, it was performed manually. Hearing thresholds were measured by increasing sound levels until there was a response from the person, and then the sound level was lowered by 10 dB and then increased by 5 dB, until a response was given once more [39]. The frequencies 500, 1000, 2000 and 4000 Hz for both ears form the basis of a mean hearing threshold in this study. Hearing loss is defined by the World Health Organization (WHO) as a mean hearing threshold of 26 dB or higher [40]. Mild hearing loss is mean hearing threshold of both ears between 26.0 dB and 40.9, moderate hearing loss is between 41.0 and 60.9 dB, and severe hearing loss is defined by mean hearing threshold of 61.0 dB or above [41].

#### Depression and anxiety symptoms

Symptoms of depression and anxiety were assessed with the “Hospital Anxiety and Depression Scale” (HADS), a self-report form with seven questions for depressive symptoms (HADS-D) and seven questions for anxiety symptoms (HADS-A) with a score range from 0–21 points of each sub-scale. A score of ≥8 on each subscale indicates clinically relevant symptoms consistent with depression (HADS-D) or anxiety (HADS-A) [42]. The HADS has been validated in Norway and found suitable for screening purposes [43].

#### Demographic and socioeconomic status

Demographic variables were gender and age (whole years) and assessment of socioeconomic status included level of education (highest completed - from primary school to university) [31].

### Statistical analysis

Data was analyzed using SPSS version 22. Group differences of the participants were described by chi-square test for categorical variables and two-sided *t*-test for continuous variables.

Unadjusted and adjusted logistic regression analyses (the Enter method, i.e. including all relevant variables simultaneously) were used to assess the main outcome of the study (to have subjective loss of hearing vs. no loss of hearing) of participants in HUNT2 with fibromyalgia versus the reference group. In addition, participants with other, widespread and localized, musculoskeletal pain were compared with the reference group. The reference group was participants without musculoskeletal pain. Men and women were analyzed separately. We adjusted for previously known confounding factors for subjective loss of hearing: measured loss of hearing (thresholds), socio-demographic factors (age and education), and psychological distress (clinical symptoms of depression and anxiety). Measured loss of hearing and age were not linearly associated with the outcome in any of the subgroups and was categorized. Two adjusted models were presented for both comparisons. Model 1 adjusted for age, education, and measured hearing thresholds. Model 2 was additionally adjusted for clinical symptoms of anxiety and depression.

Probability values less than 0.05 were considered statistically significant.

## Results

### Descriptive account of participants

The prevalence of fibromyalgia among participants who answered these questions in HUNT 2 and underwent audiometry testing was 3.3% (*N* = 1 483). The prevalence for other musculoskeletal pain was for local pain 19.7% (*N* = 8 749), for widespread pain 22.6% (*N* = 10 059), and for local and widespread pain combined 42.3% (*N* = 18 808). Respondents who had fibromyalgia or other musculoskeletal pain were more often women, in a relationship, older, with lower level of education, higher scores of anxiety and depressive symptoms and higher average hearing thresholds than the reference group without fibromyalgia and musculoskeletal pain (Table 1).

**Table 1:** Descriptive statistics

		Fibromyalgia (FM)	Musculoskeletal pain without fibromyalgia (MS)	Reference	Comparison (*P*-value)^a^	
					FM vs Ref	MS vs Ref
Total	*N* (%)	1 483 (100)	18 808 (100)	24 203 (100)		
Women	*N* (%)	1 275 (85.97)	9 948 (52.89)	11 860 (49.00)	0.000	0.000
Age	Mean (SD)	52.43 (12.02)	51.64 (15.56)	45.99 (16.77)	0.000	0.000
Single	*N* (%)	453 (30.55)	6 340 (33.71)	10 319 (42.63)	0.000	0.000
Education					0.000	0.000
Primary school	*N* (%)	716 (48.28)	7 042 (37.44)	6 647 (27.46)		
High school	*N* (%)	552 (37.22)	7 936 (42.19)	11 091 (45.82)		
Higher	*N* (%)	215 (14.50)	3 830 (20.36)	6 465 (26.71)		
Audiometry (MTH)	Mean (SD)	16.82 (13.19)	17.65 (14.80)	14.29 (13.86)	0.000	0.000
HADS-A^b^	Mean (SD)	6.38 (4.16)	4.63 (3.43)	3.57 (2.86)	0.000	0.000
HADS-D^b^	Mean (SD)	5.07 (3.65)	3.83 (3.15)	2.81 (2.67)	0.000	0.000

Reference = Ref = participants without fibromyalgia and musculoskeletal pain disorders

MTH = mean threshold of hearing based on pure tone audiometry at frequencies 0.5, 1, 2 and 4 kHz on both ears

HADS-A = self-reported questionnaire for symptoms of anxiety

HADS-D = self-reported questionnaire for symptoms of depression

^a^Descriptive comparison of categorical variables performed with chi-square and continuous variables with two-sided *t*-test

^b^Missing data from several participants on this variable. Applicable for FM, MSD and Ref

Table 2 describes the two subgroups of persons with local and widespread musculoskeletal pain other than fibromyalgia. Persons with widespread pain were more commonly women, in a relationship, older, with lower level of education and higher scores of anxious and depressive symptoms, compared to persons with local musculoskeletal pain.

**Table 2:** Local vs widespread musculoskeletal pain disorders without fibromyalgia

		Local musculoskeletal pain (LMS)	Widespread musculoskeletal pain (WMS)	Comparison LMS vs WMS (*P*-value)^a^
Total	*N* (%)	8 749 (100)	10 059 (100)	
Women	*N* (%)	4 158 (47.52)	5 790 (57.56)	0.000
Age	Mean (SD)	50.65 (16.21)	52.50 (14.91)	0.000
Single	*N* (%)	3 067 (35.06)	3 273 (32.54)	0.000
Education				0.000
Primary school	*N* (%)	2 937 (33.57)	4 105 (40.81)	
High school	*N* (%)	3 805 (43.49)	4 131 (41.07)	
Higher	*N* (%)	2 007 (22.94)	1 823 (18.12)	
Audiometry (MTH)	Mean (SD)	17.26 (14.81)	18.00 (14.78)	0.001
HADS-A^b^	Mean (SD)	4.13 (3.11)	5.07 (3.63)	0.000
HADS-D^b^	Mean (SD)	3.48 (2.97)	4.14 (3.27)	0.000

MTH = mean threshold of hearing based on pure tone audiometry at frequencies 0.5, 1, 2 and 4 kHz on both ears

HADS-A = self-reported questionnaire for symptoms of anxiety

HADS-D = self-reported questionnaire for symptoms of depression

^a^Descriptive comparison of categorical variables performed with chi-square and continuous variables with two-sided *t*-test

^b^Missing data from several participants on this variable. Applicable for FM, MSD and Ref

### The relationship between fibromyalgia and subjective hearing loss

Table 3 shows that persons with fibromyalgia had increased probability of reporting subjective hearing loss, compared to persons in the reference group. The OR (95% CI) for subjective hearing loss was 5.182 (4.278–6.277) for women and 4.368 (3.082–6.189) for men with fibromyalgia, compared to women and men in the reference group after adjustment for age, education and measured hearing thresholds (WHO grade) (model 1). After additional adjustment for clinically relevant anxious and depressive symptoms (HADS-A ≥ 8 and HADS-D ≥ 8) (model 2), the OR (95% CI) for subjective hearing loss was 4.578 (3.622–5.787) for women and 4.523 (3.077–6.647) for men with fibromyalgia.

**Table 3:** OR (95 % CI) for subjective hearing loss by fibromyalgia, socioeconomic conditions, measured hearing loss, depression and anxiety in women and men^a^

	Subjective hearing loss		Unadjusted	Adjusted for age, education and hearing loss (model 1)	In addition adjusted for anxiety and depression (model 2)
	Yes	No			
	*N* (%)	*N* (%)	OR (95% CI)	OR (95% CI)	OR (95% CI)
WOMEN (13 135)	1 085 (8.26)	12 050 (91.74)			
Fibromyalgia					
No	805 (6.79)	11 055 (93.21)	1.000 ref	1.000 ref	1.000 ref
Yes	280 (21.96)	995 (78.04)	3.865 (3.324–4.493)	5.182 (4.278–6.277)	4.578 (3.622–5.787)
Age					
20–35	89 (2.09)	4 162 (97.91)	1.000 ref	1.000 ref	1.000 ref
36–49	135 (3.36)	3 881 (96.64)	1.627 (1.240–2.133)	1.069 (0.801–1.426)	0.974 (0.713–1.329)
50–64	279 (10.24)	2 445 (89.76)	5.336 (4.182–6.809)	2.072 (1.565–2.743)	1.952 (1.437–2.651)
65+	582 (27.15)	1 562 (72.85)	17.424 (13.837–21.942)	2.354 (1.747–3.172)	2.071 (1.474–2.909)
Education					
Primary school	659 (15.45)	3 606 (84.55)	1.000 ref	1.000 ref	1.000 ref
High school	247 (4.49)	5 255 (95.51)	0.257 (0.221–0.299)	0.807 (0.665–0.980)	0.736 (0.588–0.921)
Higher	179 (5.15)	3 189 (94.69)	0.307 (0.259–0.365)	0.811 (0.658–1.000)	0.799 (0.618–1.032)
WHO hearing impairment					
None (<26 dB)	377 (3.31)	11 007 (96.69)	1.000 ref	1.000 ref	1.000 ref
Mild (26–40 dB)	325 (28.33)	822 (71.67)	11.544 (9.793–13.606)	7.859 (6.404–9.644)	8.082 (6.340–10.302)
Moderate (41–60 dB)	291 (59.75)	196 (40.25)	43.348 (35.201–53.380)	31.533 (24.444–40.678)	35.904 (26.424–48.786)
Severe (>60 dB)	92 (78.63)	25 (21.37)	107.442 (68.247–169.148)	81.067 (50.190–130.939)	90.053 (49.378–164.234)
HADS–A ≥8^b^					
No	606 (6.11)	9 312 (93.89)	1.000 ref		1.000 ref
Yes	178 (11.83)	1 327 (88.17)	2.061 (1.727–2.460)		1.732 (1.353–2.217)
HADS-D ≥8^b^					
No	786 (6.91)	10 590 (93.09)	1.000 ref		1.000 ref
Yes	155 (17.44)	734 (82.56)	2.845 (2.358–3.433)		1.131 (0.849–1.508)
Nagelkerke R^2^				0.389	0.375
−2 Log likelihood				5 056.265	3 744.466
MEN (12 551)	1 404 (11.19)	11 147 (88.81)			
Fibromyalgia					
No	1 335 (10.82)	11 008 (89.18)	1.000 ref	1.000 ref	1.000 ref
Yes	69 (33.17)	139 (66.83)	4.093 (3.050–5.493)	4.368 (3.082–6.189)	4.523 (3.077–6.647)
Age					
20–35	150 (3.93)	3 666 (96.07)	1.000 ref	1.000 ref	1.000 ref
36–49	173 (4.54)	3 638 (95.46)	1.162 (0.930–1.453)	1.007 (0.800–1.268)	0.966 (0.757–1.233)
50–64	332 (12.40)	2 346 (87.60)	3.459 (2.833–4.223)	1.692 (1.351–2.119)	1.609 (1.262–2.050)
65+	749 (33.35)	1 497 (66.65)	12.228 (10.159–14.718)	2.116 (1.661–2.695)	1.837 (1.404–2.403)
Education					
Primary school	611 (19.72)	2 487 (80.28)	1.000 ref	1.000 ref	1.000 ref
High school	505 (8.22)	5 636 (91.78)	0.365 (0.321–0.414)	0.914 (0.783–1.066)	0.886 (0.757–1.233)
Higher	288 (8.70)	3 024 (91.30)	0.388 (0.334–0.450)	0.824 (0.691–0.981)	0.765 (0.622–0.940)
WHO hearing impairment					
None (<26 dB)	438 (4.36)	9 618 (95.64)	1.000 ref	1.000 ref	1.000 ref
Mild (26–40 dB)	439 (28.16)	1 120 (71.84)	8.607 (7.437–9.961)	5.625 (4.713–6.715)	6.157 (5.054–7.500)
Moderate (41–60 dB)	410 (53.39)	358 (46.61)	25.148 (21.194–29.841)	15.120 (12.203–18.734)	17.012 (13.214–21.902)
Severe (>60 dB)	117 (69.64)	51 (30.36)	50.376 (35.766–70.955)	31.310 (21.721–45.133)	29.566 (19.085–45.802)
HADS-A ≥8^b^					
No	949 (9.20)	9 368 (90.80)	1.000 ref		1.000 ref
Yes	128 (13.43)	825 (86.57)	1.532 (1.257–1.867)		1.583 (1.225–2.044)
HADS-D ≥8^b^					
No	1 040 (9.49)	9 915 (90.51)	1.000 ref		1.000 ref
Yes	183 (21.79)	657 (78.21)	2.655 (2.227–3.166)		1.429 (1.118–1.826)
Nagelkerke R^2^				0.319	0.296
−2 Log likelihood				6 593.252	5 200.042

HADS-A = self-reported questionnaire for symptoms of anxiety in which a score of ≥8 is consistent with clinically relevant symptoms of anxiety

HADS-D = self-reported questionnaire for symptoms of depression in which a score of ≥8 is consistent with clinically relevant symptoms of depression

^a^N in this analysis ranges from 13 135 to 11 113 and 12 551 to 10 961 for respectively women and men

^b^Missing data from several participants on this variable

### The relationship between other musculoskeletal pain than fibromyalgia and subjective hearing loss

Table 4 shows that non-fibromyalgic persons who had local and widespread musculoskeletal pain had increased probability of subjective hearing loss, compared to the reference group. The probability was stronger in the subgroup with widespread musculoskeletal pain, compared to the subgroup with localized pain. The OR (95% CI) for subjective hearing loss was 1.901 (1.657–2.182) for women and 1.851 (1.662–2.061) for men with local musculoskeletal pain, and 3.145 (2.788–3.548) for women and 3.739 (3.375–4.142) for men with widespread musculoskeletal pain, compared to the reference group after adjustment for age, education and measured hearing thresholds (model 1). After additional adjustment for clinically relevant anxious and depressive symptoms (model 2), the OR (95% CI) for subjective hearing loss was 1.915 (1.627–2.255) for women and 1.796 (1.590–2.029) for men with local musculoskeletal pain, and 3.073 (2.668–3.539) for women and 3.618 (3.225–4.058) for men with widespread musculoskeletal pain.

**Table 4:** OR (95 % CI) for subjective hearing loss by musculoskeletal pain, socioeconomic conditions, measured hearing loss, depression and anxiety in women and men^a^

	Subjective hearing loss		Unadjusted	Adjusted for age, education and hearing loss (model 1)	In addition adjusted for anxiety and depression (model 2)
	Yes	No			
	*N* (%)	*N* (%)	OR (95% CI)	OR (95% CI)	OR (95% CI)
WOMEN (21 808)	2 383 (10.93)	19 425 (89.07)			
Musculoskeletal pain					
None	805 (6.79)	11 055 (93.21)	1.000 ref	1.000 ref	1.000 ref
Local	558 (13.42)	3 600 (86.58)	2.129 (1.899–2.386)	1.901 (1.657–2.182)	1.915 (1.627–2.255)
Widespread	1 020 (17.62)	4 770 (82.38)	2.937 (2.661–3.240)	3.145 (2.788–3.548)	3.073 (2.668–3.539)
Age					
20–35	156 (2.61)	5 829 (97.39)	1.000 ref	1.000 ref	1.000 ref
36–49	283 (4.22)	6 426 (95.78)	1.646 (1.349–2.007)	1.212 (0.987–1.490)	1.172 (0.940–1.460)
50–64	552 (11.21)	4 371 (88.79)	4.719 (3.934–5.661)	2.202 (1.800–2.693)	2.220 (1.785–2.762)
65+	1 392 (33.21)	2 799 (66.79)	18.583 (15.654–22.059)	2.916 (2.357–3.608)	3.064 (2.415–3.887)
Education					
Primary school	1 470 (19.31)	6 143 (80.69)	1.000 ref	1.000 ref	1.000 ref
High school	505 (5.68)	8 387 (94.32)	0.252 (0.226–0.280)	0.761 (0..665–0.870)	0.771 (0.661–0.899)
Higher	408 (7.69)	4 895 (92.31)	0.348 (0.310–0.391)	0.836 (0.726–0.964)	0.896 (0.756–1.061)
WHO hearing impairment					
None (<26 dB)	835 (4.52)	17 619 (95.48)	1.000 ref	1.000 ref	1.000 ref
Mild (26–40 dB)	723 (33.32)	1 447 (66.68)	10.543 (9.419–11.805)	6.150 (5.367–7.047)	6.393 (5.445–7.506)
Moderate (41–60 dB)	644 (67.08)	316 (32.92)	43.002 (36.959–50.035)	25.229 (21.099–30.168)	25.202 (20.300–31.286)
Severe (>60 dB)	181 (80.80)	43 (19.20)	88.819 (63.239–124.745)	57.021 (39.859–81.574)	53.218 (34.360–82.427)
HADS-A ≥8^b^					
No	1 365 (8.61)	14 493 (91.39)	1.000 ref		1.000 ref
Yes	405 (14.06)	2 476 (85.94)	1.737 (1.542–1.956)		1.703 (1.448–2.004)
HADS-D ≥8^b^					
No	1 707 (9.21)	16 830 (90.79)	1.000 ref		1.000 ref
Yes	361 (20.74)	1 379 (79.25)	2.581 (2.275–2.928)		1.285 (1.063–1.552)
Nagelkerke R^2^				0.390	0.378
−2 Log likelihood				10 334.633	7 789.274
MEN (21 203)	3 576 (16.87)	17 627 (83.13)			
Musculoskeletal pain					
None	1 335 (10.82)	11 008 (89.18)	1.000 ref	1.000 ref	1.000 ref
Local	884 (19.26)	3 707 (80.74)	1.966 (1.792–2.158)	1.851 (1.662–2.061)	1.796 (1.590–2.029)
Widespread	1 357 (31.79)	2 912 (68.21)	3.843 (3.526–4.187)	3.739 (3.375–4.142)	3.618 (3.225–4.058)
Age					
20–35	263 (5.07)	4 926 (94.93)	1.000 ref	1.000 ref	1.000 ref
36–49	489 (7.60)	5 946 (92.40)	1.540 (1.320–1.798)	1.124 (0.957–1.321)	1.064 (0.898–1.261)
50–64	1 076 (19.97)	4 311 (80.03)	4.675 (4.061–5.382)	1.978 (1.692–2.321)	1.934 (1.638–2.284)
65+	1 758 (41.70)	2 444 (58.30)	13.396 (11.665–15.385)	2.375 (2.001–2.818)	2.152 (1.784–2.597)
Education					
Primary school	1 658 (27.29)	4 418 (72.71)	1.000 ref	1.000 ref	1.000 ref
High school	1 313 (12.96)	8 822 (87.04)	0.397 (0.366–0.430)	0.892 (0.808–0.985)	0.884 (0.791–0.988)
Higher	605 (12.12)	4 387 (87.88)	0.367 (0.332–0.407)	0.757 (0.671–0.854)	0.688 (0.598–0.791)
WHO hearing impairment					
None (<26 dB)	1 222 (7.50)	15 080 (92.50)	1.000 ref	1.000 ref	1.000 ref
Mild (26–40 dB)	1 135 (37.36)	1 903 (62.64)	7.360 (6.701–8.084)	4.775 (4.270–5.341)	4.842 (4.361–5.600)
Moderate (41–60 dB)	962 (62.63)	574 (37.37)	20.682 (18.368–23.288)	12.982 (11.221–15.020)	13.428 (11.428–16.060)
Severe (>60 dB)	257 (78.59)	70 (21.41)	45.307 (34.566–59.386)	31.241 (23.419–41.677)	29.054 (20.611–40.956)
HADS-A ≥8^b^					
No	2 386 (14.25)	14 352 (85.75)	1.000 ref		1.000 ref
Yes	448 (21.13)	1 672 (78.87)	1.612 (1.440–1.804)		1.443 (1.242–1.676)
HADS-D ≥8^b^					
No	2 577 (14.39)	1 533 (85.61)	1.000 ref		1.000 ref
Yes	585 (30.02)	1 364 (69.98)	2.552 (2.296–2.836)		1.555 (1.342–1.801)
Nagelkerke R^2^				0.361	0.348
−2 Log likelihood				14 108.014	11 358.869

HADS-A = self-reported questionnaire for symptoms of anxiety in which a score of ≥8 is consistent with clinically relevant symptoms of anxiety

HADS-D = self-reported questionnaire for symptoms of depression in which a score of ≥8 is consistent with clinically relevant symptoms of depression

^a^ *N* in this analysis ranges from 21 808 to 18 210 and 21 203 to 18 355 for respectively women and men

^b^Missing data from several participants on this variable

## Discussion

In this population-based study, we found that both persons who had been diagnosed with fibromyalgia and persons with other musculoskeletal pain had increased probability for subjective hearing, compared with a reference group without fibromyalgia or other musculoskeletal pain. The findings were adjusted for gender, age, education, measured hearing impairment (audiometry, WHO graded), clinical relevant symptoms indicating anxiety and/or depression. Non-fibromyalgia respondents with widespread musculoskeletal pain had significantly higher odds to report subjective hearing loss than persons with only localized pain.

### Main findings in light of theories about central nervous sensitization

The previously mentioned theories about sustained arousal, CATS and allostatic overload all conceptualize how prolonged stress, probably in association with a genetic predisposition [44], lead to sensitization of the central nervous system, thereby enhancing the sensitivity to stimuli, by some researchers termed central sensitization syndrome (CSS) [24, 25]. This indicates that both fibromyalgia and other musculoskeletal pain might to a certain extent be explained by altered central pain processing. Central mechanisms might explain both subjective alterations in the experience of auditory stimuli and cognitive dysfunction [17]. In future studies, it would be interesting to address auditory perception among patients with fibromyalgia and other chronic pain in a prospective and nuanced manner, encompassing both experiences of explicit hearing problems and hypersensitivity to sound.

### Comparisons with previous studies

To our knowledge, our study is the first to report a relationship between subjective hearing loss and fibromyalgia, as well as for other musculoskeletal pain in a general population. The findings are in line with the previously mentioned clinical study by Wolfe et al. [28], which however did not adjust for audiometrically measured hearing loss. Hashimoto et al. [30] who revealed similar findings for conditions with three or more medical unexplained symptoms did not include fibromyalgia or other musculoskeletal pain disorders, nor did they adjust for depression and anxiety symptoms, but adjusted for measured hearing loss.

### Strengths and limitations

This study has several strengths. Firstly, despite the large sample size (over 40 000 participants) and the self-report approach used in the study, all participants were assessed with audiometry in both ears with a validated procedure. Thus, it was possible to adjust the analysis of subjective hearing loss with objectively measured hearing thresholds. Furthermore, the large sample size gave power to run subgroup analyses and adjust for a number of conditions known to affect subjective health and hearing [28, 45, 46].

Moreover, it is a strength that fibromyalgia and other musculoskeletal pain were studied separately in this study. This is because the new diagnostic criteria for fibromyalgia include more than just widespread musculoskeletal pain [18, 19]. They also include cognitive dysfunction/problems, as stated in the introduction [17, 19].

Questions concerning musculoskeletal pain have been validated through several studies [33, 34] where they compared the answers on the questionnaires against the diagnoses cervical spondylosis, adhesive capsulitis, lateral epicondylitis, carpal tunnel syndrome and Raynaud’s phenomenon [33]. Thus, the pain questions and pain map used in HUNT 2, i.e. the Nordic pain questionnaire (NPQ), appear relevant and valid [34].

The study also has some limitations. Firstly, the participants were not diagnosed by physicians in the study setting. The fibromyalgia diagnosis thus relied on the participant’s response to whether a physician prior in time had said the person had fibromyalgia. Thus, we do not know if the persons responding “yes” to fibromyalgia was evaluated using the formal diagnostic criteria. The HUNT study was conducted in 1995–1997 when the 1990 diagnostic criteria for fibromyalgia were in force, thus 2010 criteria were not used. Furthermore, we cannot rule out the possibility that participants who reported widespread pain had undiagnosed fibromyalgia. In addition, we cannot exclude that wording in the questions concerning reported subjective hearing loss influenced the responses. Furthermore, since this is a retrospective cross-sectional study, conclusions concerning causality cannot be drawn from the results.

## Conclusions

Our study showed increased probability for subjective hearing loss, both in persons with fibromyalgia and other musculoskeletal pain, especially widespread pain, after adjustment of audiometric measured hearing loss and sociodemographic and psychological variables. The finding supports the increasing recognition that medically unexplained pain conditions may pertain to a larger spectrum of symptoms, and that a common denominator for the different symptoms might be a more general dysregulation in perception of sensory stimuli.

## Abbreviations

ACR: American College of Rheumatology
CATS: Cognitive Activation Theory of Stress
CFS/ME: Chronic fatigue syndrome/myalgic encephalomyelitis
CI: Confidence intervals
CSS: Central sensitization syndromes
dB: Decibel
FM: Fibromyalgia
HADS-A/D: Hospital Anxiety and Depression Scale – Anxiety/Depression
HUNT2 Q1: HUNT2 questionnaire 1
HUNT2: The Nord-Trøndelag Health Study part 2
Hz: Hertz
IC: Interstitial cystitis
LMS: Local musculoskeletal pain
MS: Musculoskeletal pain disorders
MTH: Mean threshold of hearing
NPQ: Nordic pain questionnaire
NTHLS: The Nord-Trøndelag Hearing Loss Study
NTNU: Norwegian University of Science and Technology
OR: Odds ratio
REK: Regional Ethics Committee for Medical Research Ethics
SSS: Symptom severity score
TMD: Temporomandibular disorder
WHO: World Health Organization
WMS: Widespread musculoskeletal pain
WPI: Widespread pain index
